# An epidemic of bloody diarrhea: Escherichia coli O157 emerging in Cameroon?

**DOI:** 10.3201/eid0502.990217

**Published:** 1999

**Authors:** P Cunin, E Tedjouka, Y Germani, C Ncharre, R Bercion, J Morvan, P M Martin

**Affiliations:** Centre Pasteur du Cameroun, Yaoundé, Cameroon.

## Abstract

Between November 1997 and April 20, 1998, bloody diarrhea sickened 298 persons in Cameroon. Laboratory investigation of the epidemic (case-fatality rate, 16.4%) documented amoebiasis in one of three patients and three types of pathogens: multidrug-resistant Shigella dysenteriae type 1, S. boydii, and enterohemorrhagic Escherichia coli. We report the first isolation of E. coli O157:H7 in Cameroon and the second series of cases in the Central African region.


### An Epidemic of Bloody Diarrhea: *Escherichia coli* O157 Emerging in Cameroon?

**Patrick Cunin,* Etienne Tedjouka,† Yves Germani,‡ Chouaïbou Ncharre,§ Raymond Bercion,* Jacques Morvan,‡ and Paul M.V. Martin***

*Centre Pasteur du Cameroun, Yaoundé, Cameroon; †Lomié Health District, Lomié, Cameroon; ‡Institut Pasteur, Bangui, Central African Republic; and §World Health Organization Representation, Yaoundé, Cameroon

Between November 1997 and April 20, 1998, bloody diarrhea sickened 298 persons in Cameroon. Laboratory investigation of the epidemic (case-fatality rate, 16.4%) documented amoebiasis in one of three patients and three types of pathogens: multidrug-resistant *Shigella dysenteriae* type 1, *S. boydii,* and enterohemorrhagic *Escherichia coli.* We report the first isolation of *E. coli* O157:H7 in Cameroon and the second series of cases in the Central African region.

In December 1997, an epidemic of bloody diarrhea was reported in Ngoïla, a village of approximately 500 inhabitants, approximately 400 km from Yaoundé. Canoes and motorbikes are necessary to reach Ngoïla, which is linked to Lomié by a difficult road across the Dja River. The remote zone to the south of the river is inhabited by 2,610 persons, who belong to two ethnic groups (Bantus and Baka Pygmies) and live in 22 villages (Figure 1). The population of the outbreak area is 20,600. The sanitation system is weak, latrines do not exist, and human feces are used as fertilizer. No village has running water; drinking water, which is neither chlorinated nor filtered, comes from wells or small streams.

Between December 1997 and March 1998, teams from Lomié Hospital and the East Provincial Delegation of Health went to Ngoïla to treat patients and make an inventory of the cases. After a March 19, 1998, wire service report of a possible viral hemorrhagic fever epidemic in Ngoïla, a joint mission of Centre Pasteur du Cameroun and the World Health Organization visited the area March 25-28, 1998.

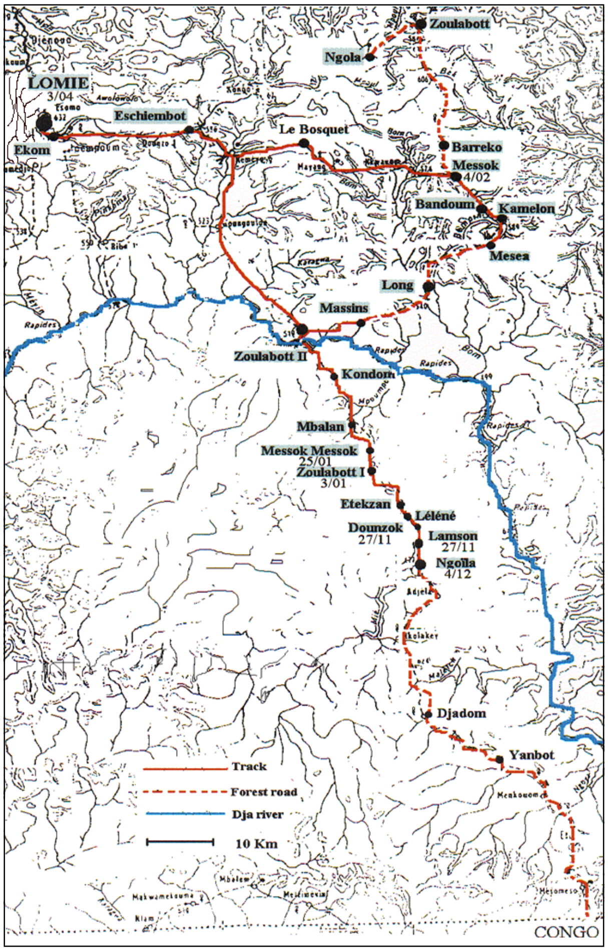

Figure 1. Progression of the bloody diarrhea epidemic, Cameroon.

Address for correspondence: Paul Martin, Centre Pasteur du Cameroun, BP 1274 Yaounde, Cameroun; fax: 237-231-564; e-mail: C.pasteur@camnet.cm.

### The Investigation

Investigators defined a case as bloody diarrhea. Data on patients' age, sex, village of origin, onset of disease, drugs received, and disease diagnosis were obtained from the observation record book of the Lomié medical officer. Standardized observation forms were filled out for 34 patients (from nine villages) seen during the mission. Serum specimens were obtained from 21 patients (both current and former) in three different villages, and stool specimens were obtained from 22 patients. Serum specimens were immediately frozen in liquid nitrogen. Stool specimens were cultured immediately in a field laboratory set up in each of the three villages. Later, in the laboratories of Centre Pasteur in Yaoundé and the Institut Pasteur of Bangui, the cultures were identified, their susceptibility to antibiotics was determined (according to the diffusion method on agar plates), and genetic studies were conducted. Hektoen agar plates, purple bromocresol agar plates, MacConkey Sorbitol medium and thiosulfate-citrate-bile- saccharose medium (Sanofi, Marnes la Coquette, France) were used.

Isolates that agglutinated in O157 antiserum were confirmed biochemically as *Escherichia coli* and screened for the lack of enzyme ß glucuronidase, using the substrate 4-methylumbelliferyl-ß-D-glucuronide. Specific anti-O157 and anti-H7 were obtained from Difco, USA. Toxin production was assayed as follows. We used the Gb3 enzyme-linked immunosorbent assay described by Ashkenazi and Cleary (1) for detection of Verotoxin. Cytotoxicity assays and seroneutralization tests were performed according to procedures (2) with Vero cells. The antisera to purified Verotoxin and Shiga toxin had been used in previous studies (3,4) and were prepared in New Zealand white rabbits to perform seroneutralization. The Verotoxin 1 preparations we used were purified from *E. coli* E40705 (O157:H7) provided by the Public Health Laboratory Service, London, United Kingdom, by the procedure described by O'Brien and LaVeck (5). Sera to Shiga toxin from *Shigella dysenteriae* type 1 (provided by the Institut Pasteur, Paris, France) were prepared according to a previously described procedure (4). Virulence genes of *E. coli* were investigated by molecular biology techniques; polymerase chain reaction was used to detect enterohemorrhagic Shigalike toxins 1 and 2 genes and the attaching and effacing gene *eae* (6-8).

Part of each stool specimen was conserved in a medium containing Merthiolate-formalized iodine for further parasitologic analyses. Viral hemorrhagic fever agents were sought by gene amplification and immunoglobulin M detection.

### The Outbreak

The first cases were reported at the end of November 1997 in two small villages, Dounzok and Lamson, near Ngoïla. An additional two cases were found retrospectively, at the end of November, one each in Djadom and Yanebot, villages south of Ngoïla. The epidemic began to affect Ngoïla at the beginning of December 1997 and then, traveling north along the roads, spread to all villages in the area. It crossed the Dja River and reached Messok on February 4 and Lomié, divisional headquarters of the district, on April 3 (Figure 1). By April 20, 298 cases had been reported from 28 villages.

In the first villages affected, each with fewer than 100 inhabitants, the epidemic lasted an average 3 months (57 days to 112 days), with an attack rate of more than 50%. Villages south of the Dja had an attack rate of 9.7% (253 of 2,610). In Ngoïla, the village with the most cases, more than a fourth of the inhabitants were sick. The graph of the epidemic for the 271 patients with a known date of illness onset suggests person-to-person transmission, with a slow increasing gradient consisting of successive waves (Figure 2).

Of 10 patients examined at Messok, 7 belonged to the same family (of 12); 2 carried *S. dysenteriae* type 1, and 3 carried enterohemorrhagic *E. coli* (EHEC). Disease onset spread out over 4 weeks, with the index

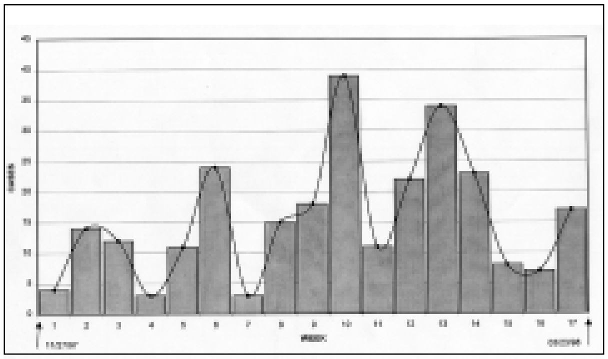

Figure 2. Cases of bloody diarrhea, by week, Cameroon, Nov. 27, 1997–March 23, 1998.

case in week 1, two cases in week 2, one case in week 3, and three cases in week 4. The median delay between illness onset was 6 days (range 3 to 8 days). A relative who came to visit family members in the Messok Health Center was the last case in this family. He became ill 7 days later; he had *Shigella* and *Entamoeba* infections. The many Pygmies in the region (who live in separate villages) were not affected by the epidemic. Both sexes in all age groups became sick. The attack rate for the villages south of the Dja (with known population sizes) was higher among female (11.4%) than among male residents (8.1%) ($p < 0.02$) and increased with age (Table 1). The case-fatality rate was highest for women ages 60 years and older (39.7%).

Table 1. Bloody diarrhea epidemic in 22 villages south of the Dja River, East Cameroon, 1997–1998

| Age group (yr) | Female | | | Male | | | p |
|---|---|---|---|---|---|---|---|
| | Cases | No.[a] | Rate (%) | Cases | No.[a] | Rate (%) | |
| 0-4 | 16 | 221 | (7.2) | 14 | 218 | (6.4) | NS[b] |
| 5-9 | 16 | 191 | (8.4) | 11 | 191 | (5.8) | NS |
| 10-19 | 25 | 321 | (7.8) | 18 | 313 | (5.8) | NS |
| 20-29 | 22 | 211 | (10.4) | 25 | 207 | (12.1) | NS |
| 30-39 | 15 | 127 | (11.8) | 14 | 154 | (9.1) | NS |
| 40-49 | 16 | 89 | (18.0) | 14 | 101 | (13.9) | NS |
| 50-59 | 11 | 63 | (17.5) | 4 | 70 | (5.7) | <0.05 |
| 60 + | 25 | 63 | (39.7) | 7 | 69 | (10.1) | $<10^{-4}$ |
| Total | 146 | 1,286 | (11.4) | 107 | 1,324 | (8.1) | <0.01 |
| p | | | $<10^{-8}$ | | | <0.05 | |

[a]Number of village inhabitants.
[b]NS, not significant.

### Illness, Treatment, and Deaths

The most frequent signs of illness in the 34 investigated patients were abdominal pain (97%) and mucus in stools (91%), followed by fever (53%), vomiting (50%), and dehydration (41%). The average number of stools per day was 10; mucus and blood were observed in stools starting on the third day. The quantity of blood in the stools varied; occasionally only blood was excreted.

Stool specimens were obtained from 12 female and 10 male patients; their median age was 25 (2 years to 60 years), and the median length of illness was 7 days (3 days to 58 days). African hemorrhagic fever was not detected. Parasitologic examination of stool specimens showed heavy infestation with numerous parasites. Hematophagous trophozoites of *Entamoeba histolytica histolytica* were observed in 7 (31.8%) of 22 of the cases. Bacteriologic studies isolated at least 1 enteropathogen in 20 (90.9%). EHEC were isolated in 12 (54.5%), *S. dysenteriae* type 1 in 9 (40.9%), and *S. boydii* in 2 (9.1%). Potentially dangerous associations were common. Of the 22 patients with stool specimens, 7 (31.8%) had both *E. histolytica histolytica* and one enteropathogenic bacterium; 3 (13.6%) had both *E. histolytica histolytica* and *S. dysenteriae* type 1; and 2 (9.1%) had both *S. dysenteriae* type 1 and EHEC. Simultaneous infection with all three pathogens—*S. dysenteriae*, EHEC, and *E. histolytica*—were found in patients from all three villages, while dual infections with *S. dysenteriae* and EHEC were observed in patients from two villages.

Drug susceptibility testing found that *S. dysenteriae* type 1 was resistant to amoxicillin, amoxicillin in combination with clavulanic acid, tetracycline, chloramphenicol, cefsulodin, and cotrimoxazole; it was sensitive to nalidixic acid, piperacillin, cefalotin, ceftazidin, gentamicin, and ofloxacin. *E. coli* O157:H7 was resistant to amoxicillin, chloramphenicol, and cefsulodine, and sensitive to amoxicillin in combination with clavulanic acid, tetracycline, cotrimoxazole, nalidixic acid, and cefalotin. DNA amplification showed that *E. coli* O157:H7 strains had genes that coded for Shigalike toxins 1 and 2 and the attaching and effacing gene *eaeA*.

By April 1998, five different medications, depending on availability, had been used, sometimes in combination. Of the 292 patients with known drug regimens, 182 took cotrimoxazole (62.3%), 190 took metronidazole (65.1%), 34 took chloramphenicol (11.6%), and 19 took tetracycline (6.5%). Of these, 67 (22.9%) were given oral rehydration salts, while 71 (24.3%) received no treatment or received traditional medicine. More female than male patients were not treated, 51 (31.1%) of 164 versus 20 (15.6%) of 128 ($p < 0.01$), respectively. The groups that received the least or no drug treatment were children younger than 5 years of age (12 [34.3%] of 35) and those ages 50 years and older (26 [46.4%] of 56). The proportion of patients treated with drugs increased during the epidemic: 24 (54.5%) of 44 of the patients were treated in December 1997, while 41 (68.3%) of 60, 85 (76.6%) of 111, and 44 (88%) of 50 were treated in January, February, and March, 1998, respectively.

Of the 275 patients for whom disease outcome was known, 45 died (case-fatality rate

16.4%). The rate was higher in female (21.3% [33 of 155]) than in male patients, (10% [12 of 120]) (p <0.02) (Table 2). Case-fatality rates were 23.5% (8 of 34) among those younger than 5 years old, 40% (22 of 55) among those 50 years of age and older, and 53.5% (38 of 71) among patients who received no treatment.

Table 2. Bloody diarrhea epidemic in East Cameroon, case-fatality rate of by age and sex

| Age group | Female | | | Male | | | |
|---|---|---|---|---|---|---|---|
| (yr) | Cases | Deaths | (%) | Cases | Deaths | (%) | p |
| 0-4 | 18 | 5 | (27.8) | 16 | 3 | (18.8) | NS[a] |
| 5-9 | 16 | 2 | (12.5) | 13 | 1 | (7.7) | NS |
| 10-19 | 25 | 1 | (4.0) | 20 | 1 | (5.0) | NS |
| 20-29 | 24 | 1 | (4.2) | 27 | 1 | (3.7) | NS |
| 30-39 | 16 | 3 | (18.8) | 18 | 1 | (5.6) | NS |
| 40-49 | 17 | 3 | (17.6) | 12 | 1 | (8.3) | NS |
| 50-59 | 13 | 6 | (46.2) | 8 | 2 | (25.0) | NS |
| 60 + | 26 | 12 | (46.2) | 6 | 2 | (33.3) | NS |
| Total | 155 | 33 | (21.3) | 120 | 12 | (10.0) | <0.02 |

[a]NS, not significant.

### Conclusions

During the first half of 1997, an epidemic of bloody diarrhea with a case-fatality rate of 19% was reported in villages near Mintom, a district headquarters approximately 100 km west of Ngoïla. Then, the region of Ngoïla in the East Province of Cameroon was hit by an extremely severe and deadly epidemic of bloody diarrhea, which continued until July 1998. In April 1998, 34 (11.4%) of 298 (the affected population) were examined, and stool specimens were obtained from 22 patients with an active infection, which represented 7.4% of the total epidemic population (298). Although the sample was relatively small, three bacterial enteropathogens (*S. dysenteriae* type 1, *S. boydii,* and EHEC) associated with numerous parasites were isolated from the stool specimens. Thus, which of these pathogens were responsible for the epidemic and its severity is not known with certainty. The isolation of *E. coli* O157, in contrast to *Shigella,* is quite recent in Africa. We believe that this is the first case of *E. coli* O157 described in Cameroon and the second (after the 1996 epidemic in the Republic of Central Africa) in the Central African region (9).

A few persons may have contracted the disease through food or drink. However, the epidemic curve with a slow rise of successive waves does not depict a common source of contamination; it suggests person-to-person transmission. The spread of the disease followed roads, and women were the most likely infected, probably because they live together in groups and take care of the sick. Because of difficulties in communication and the necessity to return to the laboratory to process the specimens, we could not investigate in detail with a case-control study. The protracted course of the illness, poor sanitation, and length of time patients were infected with pathogens favored transmission. The bloody diarrhea was long-lasting for some patients. In one patient with *S. dysenteriae* type I, the onset of disease had begun 58 days before, while in another with EHEC, it had begun 39 days before.

In contrast to shigellosis, which is most often transmitted from person to person (10) (often through the hands) (11) and affects mostly women (12), *E. coli* O157 infection has been described in the northern hemisphere as food poisoning (13). For example, in Japan an epidemic involving more than 5,000 cases was described in school children contaminated by food prepared in the school canteen in 1996 (14). Sources of contamination most commonly incriminated are meat in hamburgers (15) or sandwiches (16), milk (17), drinking water (18), water absorbed during baths (19), nonpasteurized apple juice (20), and salads (21). The main reservoir of the germ is cows and other ruminants (22), but the association between *E. coli* O157 and cows is not absolute (23). Its transmission from person to person has been observed in a small number of cases, most often intrafamilially (24). In the Republic of Central Africa during the first isolation of *E. coli* O157, food was the suspected vehicle (9). The rain forest zone in South and East Cameroon, like Ngoïla, has no cattle; breeding is limited to pigs, goats, and chickens; and meat is not imported.

Deaths caused by the disease were quite high, with a case-fatality rate of 16.4%. In comparison, the case-fatality rate for cholera was globally estimated at 4.7% in 1996 (25) and rose to 48% in the worst outbreak (the beginning of the epidemic in Rwanda refugee camps in Goma in 1994) (26). Shigellosis is one of the main causes of severe diarrhea in Africa, accounting for 12% of all deaths in Kibue Sector in Burundi in 1992 (12) and 19% of pediatric hospital deaths in KwaZulu-Natal in 1995 (27). The portion of deaths due to shigellosis varies—from 13% in hospital statistics in children in KwaZulu-Natal to 3.8% in a Burundan refugee camp in Rwanda in 1993 (28) and 0.25% during the first *Shigella* epidemic in Mozambique in the same year (29). The severity of *E. coli* O157 is associated with its complication, hemolytic uremic syndrome; an estimated 200,000 cases of this syndrome per year occur in the United States, with 250 deaths and a case-fatality rate of 0.1% (30). In the epidemic in the Republic of Central Africa, the case-fatality rate was 3.7% (9).

The causes of death were unknown. The case-fatality rate diminished during the course of the epidemic: 14 (31.8%) of 44 in patients in December 1997, 9 (15%) of 60 in January, 19 (18.3%) of 104 in February, and 2 (5%) of 40 in March 1998 ($p < 0.02$), suggesting progressively better disease management during the epidemic. The case-fatality rate was 3.4% in patients who received either cotrimoxazole (6/174), metronidazole (6/176), or chloramphenicol (1/29); 5.5% in those who were given tetracycline (1/18); 0% in patients who also received oral rehydration salts; and 53.5% (38/71) in those who received no treatment. In Ngoïla, health facilities did not have the resources to perform the classic hematologic and chemical investigations. However, the case-fatality rate in patients who received oral rehydration salts was zero, suggesting that fluid loss was a major part of the disease. Other cofactors (e.g., anemia) could have played a role in lethality. To determine the risk for death from the disease, we constructed a logistic regression model that included age, sex, onset of illness, and drug regimen received. Oral rehydration salts were not included in the model (all rehydrated patients survived). The only factor strongly associated with death was age >50 years (odds ratio [OR] = 5 ; 95% confidence interval [CI]: 2.2-11.5); having received cotrimoxazole was a protective factor (OR = 0.05, 95% CI: 0.02 - 0.13, $p < 10^{-5}$). Because the treatment groups were not randomized, the decline in case-fatality rates cannot be attributed to this drug. The use of other drugs, however, as well as sex and month of onset of the illness, were not associated with death. *S. dysenteriae* type 1 was resistant to cotrimoxazole, while *E. coli* O157 was susceptible.

The multiple resistance to antibiotics of *S. dysenteriae* type 1 strains isolated from Ngoïla could also explain the high case-fatality rate. Studies from Central and East Africa confirm that *S. dysenteriae* type 1 is resistant to multiple drugs (31-34). There may, therefore, be a new emerging disease due to the association of enterohemorrhagic *E. coli* O157:H7 and *S. dysenteriae* type 1 in this region of Africa, an association that could be as deadly as that of shigellosis and cholera. Finally, the management of cases will be rendered difficult by the resistance of the pathogens implicated to multiple drugs. After our microbiologic investigations, the health authorities distributed nalidixic tablets and ciprofloxacin. The case-fatality rate fell to 3.7% (8/213).

Dr. Cunin, an epidemiologist and specialist in tropical diseases, is affiliated with Coopération française and has worked in Africa (Morocco, Côte d'Ivoire, Mauritania, Cameroon) since 1975. He now works at the Pasteur Center in Cameroon. His research focuses on epidemic investigation, infectious diseases (tuberculosis, arbovirus, schistosomiasis), mother-to-child transmission of HIV, and maternal and child health.

### References

1. Askenazi S, Cleary TG. Rapid method to detect Shiga toxin and Shiga-like toxin I based on binding to globotriosyl ceramide (Gb3), their natural receptor. J Clin Microbiol 1989;27:1145-50.
2. Marques LRM, Moore MA, Wells JC, Waschmuth IK, O'Brien AD. Production of Shiga-like toxin by *Escherichia coli*. J Infect Dis 1986;154:338-41.
3. Germani Y, Morillon M, Begaud E, Dubourdieu H, Costa R, Thevenon J. Two-year study of endemic enteric pathogens associated with acute diarrhea in New Caledonia. J Clin Microbiol 1994;32:1532-6.
4. Germani Y, Begaud E, Desperrier JM. Easy-to-perform modified Elek test to identify Shiga-like toxin-producing diarrhoeogenic *Escherichia coli*. Res Microbiol 1994;145:333-40.
5. O'Brien AD, LaVeck GD. Purification and characterization of a Shigella dysenteriae 1-like toxin produced by *Escherichia coli*. Infect Immun 1985;40:675-83.
6. Germani Y, Begaud E, Le Bouguenec C. Detection of *Escherichia coli* attaching and effacing gene (eaeA) in enteropathogenic strains by polymerase chain reaction. Res Microbiol 1997;148:177-81.
7. Pollard DR, Johnson WM, Lior H, Tyler SD, Rozee R. Rapid and specific detection of verotoxin genes in *Escherichia coli* by the polymerase chain reaction. J Clin Microbiol 1990;28:540-5.
8. Pollard DR, Johnson WM, Lior H, Tyler SD, Rozee R. Identification of verotoxin 2 variant B subunit genes in *Escherichia coli* by polymerase chain reaction and restriction fragment length polymorphism analysis. J Clin Microbiol 1991;29:1339-43.
9. Germani Y, Soro B, Vohito M, Morel O, Morvan J. Enterohaemorrhagic *Escherichia coli* in Central African Republic. Lancet 1997;349:1670.

10. Shears P. Shigella infections. Ann Trop Med Parasitol 1996;90:105-14.
11. Tuttle J, Ries AA, Chimba RM, Perera CU, Bean NH, Griffin PM. Antimicrobial-resistant epidemic *Shigella dysenteriae* type 1 in Zambia: modes of transmission. J Infect Dis 1995;171:371.
12. Birmingham ME, Lee LA, Ntakibirora M, Bizimana F, Deming MS. A household survey of dysentery in Burundi: implications for the current pandemic in sub-Saharan Africa. Bull World Health Organ 1997;75:45-53.
13. Takeda Y. Enterohaemorrhagic *Escherichia coli*. World Health Stat Q 1997;50:74-80.
14. Kawamura T. The clinical course and laboratory data in haemorrhagic colitis caused by *Escherichia coli* O157:H7. Japanese Journal of Clinical Pathology 1997;45:865-8.
15. Cieslak PR, Noble SJ, Maxson DJ, Empey LC, Ravenholt O, Legarza G, et al. Hamburger-associated *Escherichia coli* O157:H7 infection in Las Vegas: a hidden epidemic. Am J Public Health 1997;87:176-80.
16. McDonnell RJ, Rampling A, Crook S, Cockcroft PM, Wilshaw GA, Cheasty T, et al. An outbreak of Vero cytotoxin producing *Escherichia coli* O157 infection associated with takeaway sandwiches. Commun Dis Rep CDR Rev 1997;7:R201-5.
17. Bielaszewska M, Janda J, Blahova K, Minarikova H, Jikova E, Karmali MA, et al. Human *Escherichia coli* O157:H7 infection associated with consumption of unpasteurised goat's milk. Epidemiol Infect 1997;119:299-305.
18. Jones IG, Roworth M. An outbreak of *Escherichia coli* O157 and campylobacteriosis associated with contamination of a drinking water supply. Public Health 1996;110:277-82.
19. Ackman D, Marks S, Mack P, Caldwell M, Root T, Birkhead G. Swimming-associated haemorrhagic colitis due to *Escherichia coli* O157:H7 infection: evidence of prolonged contamination of a fresh water lake. Epidemiol Infect 1997;119:1-8.
20. Outbreaks of *Escherichia coli* O157:H7 infection and cryptosporidiosis associated with drinking unpasteurised apple cider. Connecticut and New York, October 1996 [editorial]. JAMA 1997;277:781-2.
21. Watanabe Y, Ozasa K. An epidemiological study on an outbreak of *Escherichia coli* O157:H7 infection. Japanese Journal of Clinical Pathology 1997;45:869-74.
22. Allerberger F, Solder B, Caprioli A, Karch H. Enterohaemorrhagic *Escherichia coli* and hemolytic-uremic syndrome. Wien Klin Wochenschr 1997;109:669-77.
23. Chart H. Are all infections with *Escherichia coli* O157 associated with cattle? Lancet 1998;352:1005.
24. Judwig K, Ruder H, Bitzan M, Zimmermann S, Karch H. Outbreak of *Escherichia coli* O157:H7 infection in a large family. Eur J Clin Microbiol Infect Dis 1997;16:238-41.
25. Cholera in 1996. Wkly Epidemiol Rec 1997;72:229-35.
26. Siddike AK, Salam A, Islam MS, Akram K, Majumdar RN, Zaman K, et al. Why treatment centers failed to prevent cholera deaths among Rwandan refugees in Goma, Zaire. Lancet 1995;345:359-61.
27. Chopra M, Wilkinson D, Stirling S. Epidemic shigella dysentery in children in northern KwaZulu-Natal. S Afr Med J 1997;87:48-51.
28. Paquet C, Leborgne P, Sasse A, Varaine F. An outbreak of *Shigella dysenteriae* type 1 in a refugee camp in Rwanda. Sante 1995;5:181-4.
29. Aragon M, Barreto A, Chambule J, Noya A, Tallarico M. Shigellosis in Mozambique: the 1993 outbreak rehabilitation—a follow up study. Trop Doct 1995;25:159-62.
30. Koutkia P, Mylonakis E, Flanigan T. Enterohemorrhagic *Escherichia coli* O157:H7—an emerging pathogen. Am Fam Physician 1997;56:853-6.
31. Ndihokubwayo JB, Baribwira C, Ndayiragije A, Poste B. Antibiotic sensitivity of 299 strains of *Shigella* isolated in Burundi. Med Trop (Mars) 1996;56:37-40.
32. Engels D, Madaras T, Nyanndwi S, Murray J. Epidemic dysentery caused by *Shigella dysenteriae* type 1: a sentinel site surveillance of antimicrobial resistance patterns in Burundi. Bull World Health Organ 1995;73:787-91.
33. Bogaerts J, Verhaegen J, Munyabikali JP, Mukantabana B, Lemmens P, Vandeven J, et al. J. Antimicrobial resistance and serotypes of *Shigella* isolates in Kigali, Rwanda (1983 to 1993): increasing frequency of multiple resistance. Diagn Microbiol Infect Dis 1997;28:165-71.
34. Cavallo JD, Bercion R, Baudet J-M, Samson T, France M, Meyran M. Étude de la sensibilité aux antibiotiques de 140 souches de Shigelles isolées à Djibouti. Bull Soc Pathol Exot 1993;86:35-40.
